# Conditional survival analysis and real-time prognosis prediction in stage III T3–T4 colon cancer patients after surgical resection: a SEER database analysis

**DOI:** 10.1007/s00384-024-04614-x

**Published:** 2024-04-19

**Authors:** Hao Zeng, Xueyi Xue, Dongbo Chen, Biaohui Zheng, Baofeng Liang, Zhipeng Que, Dongbo Xu, Xiaojie Wang, Shuangming Lin

**Affiliations:** 1 Department of Gastroenterology and Anorectal Surgery, Longyan First Affiliated Hospital of Fujian Medical University, Longyan, China; 2 Department of Gastroenterology and Anorectal Surgery, Longyan First Hospital, Fujian Medical University, No. 105 Jiuyi North Road, Longyan, 364000 Fujian Province China; 3 Department of Surgery II, Shanghang County Hospital, Longyan City, Fujian Province China; 4 Department of Colorectal Surgery, Union Hospital, Fujian Medical University, No. 29 Xinquan Road, Fuzhou, 350001 Fujian Province China

**Keywords:** Stage III T3–T4 colon cancer, Conditional overall survival, Conditional cancer-specific survival, Nomogram, Surveillance, Epidemiology, and End Results

## Abstract

**Background:**

Conditional survival (CS) takes into consideration the duration of survival post-surgery and can provide valuable additional insights. The aim of this study was to investigate the risk factors associated with reduced one-year postoperative conditional survival in patients diagnosed with stage III T3–T4 colon cancer and real-time prognosis prediction. Furthermore, we aim to develop pertinent nomograms and predictive models.

**Methods:**

Clinical data and survival outcomes of patients diagnosed with stage III T3–T4 colon cancer were obtained from the Surveillance, Epidemiology, and End Results (SEER) database, covering the period from 2010 to 2019. Patients were divided into training and validation cohorts at a ratio of 7:3. The training set consisted of a total of 11,386 patients for conditional overall survival (cOS) and 11,800 patients for conditional cancer-specific survival (cCSS), while the validation set comprised 4876 patients for cOS and 5055 patients for cCSS. Univariate and multivariate Cox regression analyses were employed to identify independent risk factors influencing one-year postoperative cOS and cCSS. Subsequently, predictive nomograms for cOS and cCSS at 2-year, 3-year, 4-year, and 5-year intervals were constructed based on the identified prognostic factors. The performance of these nomograms was rigorously assessed through metrics including the concordance index (*C*-index), calibration curves, and the area under curve (AUC) derived from the receiver operating characteristic (ROC) analysis. Clinical utility was further evaluated using decision curve analysis (DCA).

**Results:**

A total of 18,190 patients diagnosed with stage III T3–T4 colon cancer were included in this study. Independent risk factors for one-year postoperative cOS and cCSS included age, pT stage, pN stage, pretreatment carcinoembryonic antigen (CEA) levels, receipt of chemotherapy, perineural invasion (PNI), presence of tumor deposits, the number of harvested lymph nodes, and marital status. Sex and tumor site were significantly associated with one-year postoperative cOS, while radiation therapy was notably associated with one-year postoperative cCSS. In the training cohort, the developed nomogram demonstrated a C-index of 0.701 (95% CI, 0.711–0.691) for predicting one-year postoperative cOS and 0.701 (95% CI, 0.713–0.689) for one-year postoperative cCSS. Following validation, the C-index remained robust at 0.707 (95% CI, 0.721–0.693) for one-year postoperative cOS and 0.700 (95% CI, 0.716–0.684) for one-year postoperative cCSS. ROC and calibration curves provided evidence of the model's stability and reliability. Furthermore, DCA underscored the nomogram’s superior clinical utility.

**Conclusions:**

Our study developed nomograms and predictive models for postoperative stage III survival in T3–T4 colon cancer with the aim of accurately estimating conditional survival. Survival bias in our analyses may lead to overestimation of survival outcomes, which may limit the applicability of our findings.

## Introduction

Stage III colon cancer is characterized by lymph node metastases, and T3–T4 stage tumors usually deeply infiltrate the colon wall into the pericolonic tissues and nearby lymph nodes, implying a greater tumor load and deeper infiltration [1]. In stage III colorectal cancer, the proportion of T3–T4 stage tumors is as high as 84.3% or 91.6% [2]. Due to the deeper depth of invasion of these tumors, the prognosis is usually poorer and the risk of local and distant recurrence is higher [3]. Numerous studies have consistently emphasized that lymph node involvement is a key determinant of colorectal cancer progression and prognosis [4]. Despite significant advances in the clinical management of stage III colon cancer, our understanding of how survival evolves over time in patients with stage III T3–T4 colon cancer remains relatively limited.

While many survival rates reported in the literature are static and calculated from the date of diagnosis or surgery, assuming a uniform distribution of postoperative mortality or recurrence risk [5], recent research indicates that the risk of postoperative mortality or recurrence varies over time [6]. Consequently, for long-term survivors, assessing prognosis solely at the baseline underestimates the dynamic changes in survival. This approach often leads to frequent follow-up monitoring and an increased psychological burden for patients. Conditional survival (CS) addresses this issue by estimating the probability of survival for a specific number of years following diagnosis or treatment while taking into account the time the patient has survived. As a result, CS offers a more personalized prognosis over a defined period, facilitating the adaptation of postoperative follow-up strategies.

In addition to the postoperative duration, factors such as tumor-node-metastasis (TNM) staging and tumor size significantly influence patient prognosis. Studies have demonstrated that survival nomograms, which incorporate multiple critical prognostic factors, serve as precise tools for evaluating postoperative survival [1, 7]. While several survival nomograms have been developed for colon cancer patients thus far, it is worth noting that these models often give limited consideration to the patient’s postoperative survival time

Therefore, the purpose of this study is to utilize data extracted from the Surveillance, Epidemiology, and End Results (SEER) database to identify risk factors associated with reduced conditional survival rates one year after surgery in patients with stage III T3–T4 colon cancer. Our objective is to evaluate conditional survival, including conditional overall survival (cOS) and conditional cancer-specific survival (cCSS), following curative surgery. Additionally, we intend to develop conditional survival nomograms for predicting conditional survival probabilities following the resection of stage III T3–T4 colon cancer.

## Materials and methods

### Included participants

This retrospective cohort study utilized data from patients diagnosed and pathologically confirmed as stage III T3–T4 colon cancer (limited to those with a single primary tumor) extracted from a total of 18 registries using the National Cancer Institute’s SEER Cancer database for the period 2010 to 2019. Data screening and retrieval were conducted using SEER*Stat 8.4.2 software (http://seer.cancer.gov/seerstat/). Eligible patients were selected based on the following inclusion criteria: (1) diagnosis of stage III T3–T4 colon cancer according to the International Classification of Diseases for Oncology, Third Edition (ICD-O-3), histology codes 8140–8389 (adenocarcinomas); (2) diagnosis date falling within the range of 2010 to 2019; and (3) availability of active follow-up data with well-defined causes of mortality for deceased patients. Exclusion criteria encompassed patients with non-primary tumors, unclear pathological diagnoses, invalid follow-up data, appendiceal tumors or unclear tumor locations, unclear pathological grades, unspecified tumor sizes, uncertain numbers of harvested lymph nodes, or unclear tumor grades as per the AJCC classification (8th version). For each patient, the study collected the following information: age, sex, race, tumor stage, histological grade, tumor site, tumor size, number of harvested lymph nodes, scope of regional lymph nodes, marital status, pretreatment carcinoembryonic antigen (CEA) levels, perineural invasion (PNI), receipt of postoperative chemotherapy/radiation, presence of tumor deposits, survival time in months, and survival status.

### Data extraction

**Fig. 1:**
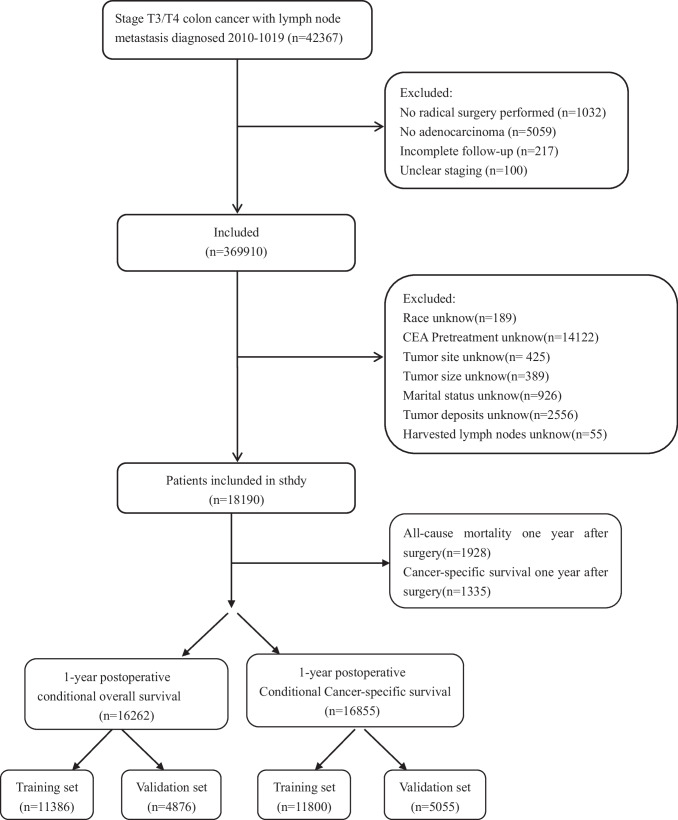
Study flow chart

Patients were divided into training and validation cohorts at a ratio of 7:3. The training set consisted of a total of 11,386 patients for cOS and 11,800 patients for cCSS, while the validation set comprised 4876 patients for cOS and 5055 patients for cCSS (Fig. 1). Marital status was categorized as either married or unmarried (single, widowed, divorced, and separated). The number of sampled lymph nodes was grouped as < 12 or ≥ 12, and tumor size was categorized as < 5 cm or ≥ 5 cm using the X-tile program [8].

### Statistical analysis

Overall survival (OS) was calculated from the time of bowel resection until death from any cause or the last follow-up visit. Cancer-specific survival (CSS) was defined as the time between the date of surgery and the date of recurrence, or last follow-up if there was no recurrence. Conditional survival (CS) is defined as the probability of surviving for another *y* years if the patient has survived for *x* years and can be calculated from Kaplan–Meier survival data. In this study, we evaluated cOS and cCSS. The mathematical expression for CS is CS(*x*|*y*) = *S*(*x* + *y*) / *S*(*x*), where *S*(*x*) represents the survival estimate calculated using the Kaplan–Meier method at x years after surgery [9]. For example, cOS(2|3) represents the probability that a patient who has survived 2 years will survive another 3 years, calculated by dividing the 5-year Kaplan–Meier overall survival estimate (OS(5)) by the 2-year overall survival estimate (OS(2)) [9, 10].

All patients were randomly allocated to either the training or validation cohorts using a 7:3 ratio. The primary outcome measures for this study included one-year postoperative cOS and cCSS [11]. Categorical variables were presented as numbers and percentages (*n*, %), and differences in variable distribution between the training and validation cohorts were assessed using the chi-square test. Variables with a *P*-value < 0.05 in the univariate analysis were subsequently included in the multivariate analysis. A multivariate Cox proportional hazards model, employing backward elimination, was employed to derive the most acolon cancerurate and parsimonious model for identifying survival predictors. The assumptions underlying the Cox proportional hazards model were assessed and found to be met. Based on the predictive model utilizing the identified prognostic factors, CS nomograms were constructed to predict the 2-year, 3-year, 4-year, and 5-year postoperative cOS and cCSS [12].

The performance of the nomogram in both the training and validation cohorts was assessed through the following steps: Concordance index (*C*-index) was employed to evaluate the predictive performance of the nomogram. The area under the receiver operating characteristic (ROC) curve (AUC) with a 95% confidence interval (CI) was calculated to assess the nomogram's discrimination ability. An AUC value exceeding 0.7 was considered indicative of good predictive capabilities [13]. Additionally, decision curve analysis (DCA) was conducted to compare the clinical utility of the nomogram. All statistical analyses were carried out using R software (version 4.3.1), and a two-sided *P*-value < 0.05 was considered statistically significant.

## Results

### Basic characteristics of the patients

The demographic and clinical characteristics of stage III T3–T4 colon cancer patients in both the training and validation cohorts are summarized in Table 1. With the exception of perineural invasion, no significant differences in demographic and clinical characteristics were observed between the training and validation groups.

**Table 1:** Baseline characteristics of the training set and validation set based on 1-year postoperative conditional overall survival and conditional cancer-specific survival

	**1-year postoperative conditional overall survival**			**1-year postoperative conditional cancer-specific survival**		
	**Training set** **(*N* = 11,386)**	**Validation set** **(*N* = 4876)**	***P*-value**	**Training set** **(*N* = 11,800)**	**Validation set** **(*N* = 5055)**	***P*-value**
**Age**						
< 50 years	1593 (14.0%)	714 (14.6%)	0.328	1601 (13.6%)	721 (14.3%)	0.58
50–59–years	2330 (20.5%)	1001 (20.5%)		2379 (20.2%)	992 (19.6%)	
60–69–years	2984 (26.2%)	1323 (27.1%)		3068 (26.0%)	1340 (26.5%)	
70–79–years	2584 (22.7%)	1069 (21.9%)		2704 (22.9%)	1123 (22.2%)	
80+ years	1895 (16.6%)	769 (15.8%)		2048 (17.4%)	879 (17.4%)	
**Sex**						
Female	5531 (48.6%)	2415 (49.5%)	0.274	5746 (48.7%)	2469 (48.8%)	0.874
Male	5855 (51.4%)	2461 (50.5%)		6054 (51.3%)	2586 (51.2%)	
**Race**						
American Indian/Alaska Native	91 (0.8%)	43 (0.9%)	0.822	90 (0.8%)	47 (0.9%)	0.352
Asian or Pacific Islander	1296 (11.4%)	543 (11.1%)		1293 (11.0%)	586 (11.6%)	
Black	1396 (12.3%)	617 (12.7%)		1479 (12.5%)	608 (12.0%)	
White	8603 (75.6%)	3673 (75.3%)		8938 (75.7%)	3814 (75.5%)	
**Site**						
Ascending colon	2060 (18.1%)	889 (18.2%)	0.866	2166 (18.4%)	929 (18.4%)	0.061
Cecum	2428 (21.3%)	1014 (20.8%)		2539 (21.5%)	1045 (20.7%)	
Descending colon	759 (6.7%)	315 (6.5%)		766 (6.5%)	337 (6.7%)	
Hepatic flexure	522 (4.6%)	234 (4.8%)		520 (4.4%)	262 (5.2%)	
Rectosigmoid junction	1160 (10.2%)	490 (10.0%)		1170 (9.9%)	528 (10.4%)	
Sigmoid colon	3072 (27.0%)	1321 (27.1%)		3213 (27.2%)	1300 (25.7%)	
Splenic flexure	393 (3.5%)	191 (3.9%)		404 (3.4%)	202 (4.0%)	
Transverse colon	992 (8.7%)	422 (8.7%)		1022 (8.7%)	452 (8.9%)	
**pT**						
T3	8755 (76.9%)	3747 (76.8%)	0.963	9050 (76.7%)	3866 (76.5%)	0.585
T4a	1860 (16.3%)	803 (16.5%)		1958 (16.6%)	828 (16.4%)	
T4b	771 (6.8%)	326 (6.7%)		792 (6.7%)	361 (7.1%)	
**pN**						
N1a	3550 (31.2%)	1470 (30.1%)	0.577	3629 (30.8%)	3629 (30.8%)	0.993
N1b	3606 (31.7%)	1598 (32.8%)		3783 (32.1%)	1614 (31.9%)	
N1c	570 (5.0%)	234 (4.8%)		592 (5.0%)	249 (4.9%)	
N2a	2162 (19.0%)	937 (19.2%)		2247 (19.0%)	954 (18.9%)	
N2b	1498 (13.2%)	637 (13.1%)		1549 (13.1%)	670 (13.3%)	
**Scope of regional lymph nodes**						
1 to 3 regional LNs	110 (1.0%)	64 (1.3%)	0.144	133 (1.1%)	49 (1.0%)	0.658
4 or more regional LNs	11,160 (98.0%)	4763 (97.7%)		11,548 (97.9%)	4954 (98.0%)	
None	116 (1.0%)	49 (1.0%)		119 (1.0%)	52 (1.0%)	
**Radiation**						
None/unknown	10,899 (95.7%)	4670 (95.8%)	0.913	11,327 (96.0%)	4823 (95.4%)	0.092
Yes	487 (4.3%)	206 (4.2%)		473 (4.0%)	232 (4.6%)	
**Chemotherapy**						
No/unknown	3002 (26.4%)	1306 (26.8%)	0.593	3345 (28.3%)	1413 (28.0%)	0.615
Yes	8384 (73.6%)	3570 (73.2%)		8455 (71.7%)	8455 (71.7%)	
**CEA pretreatment**						
CEA negative/normal	6462 (56.8%)	2698 (55.3%)	0.097	6617 (56.1%)	2821 (55.8%)	0.759
CEA positive/elevated	4924 (43.2%)	2178 (44.7%)		5183 (43.9%)	2234 (44.2%)	
**Harvested lymph nodes**						
< 12 LNs	828 (7.3%)	389 (8.0%)	0.125	900 (7.6%)	388 (7.7%)	0.939
≥ 12 LNs	10,558 (92.7%)	4487 (92.0%)		10,900 (92.4%)	4667 (92.3%)	
**Tumor deposits**						
No	9140 (80.3%)	3941 (80.8%)	0.43	9461 (80.2%)	4091 (80.9%)	0.269
Yes	2246 (19.7%)	935 (19.2%)		2339 (19.8%)	964 (19.1%)	
**Tumor size**						
< 5 cm	6080 (53.4%)	2586 (53.0%)	0.683	6269 (53.1%)	2673 (52.9%)	0.78
≥ 5 cm	5306 (46.6%)	2290 (47.0%)		5531 (46.9%)	2382 (47.1%)	
**Perineural invasion**						
No/unknown	9212 (80.9%)	4021 (82.5%)	0.02	9573 (81.1%)	4134 (81.8%)	0.329
Yes	2174 (19.1%)	855 (17.5%)		2227 (18.9%)	921 (18.2%)	
**Marital status**						
Married	6437 (56.5%)	2741 (56.2%)	0.719	6628 (56.2%)	2814 (55.7%)	0.559
Unmarried	4949 (43.5%)	2135 (43.8%)		5172 (43.8%)	2241 (44.3%)	

*pT* pathologic Tumor, *pN* pathologic Nodes, *LNs* Lymph Nodes, *CEA* carcinoembryonic antigen

### Conditional survival

**Fig. 2:**
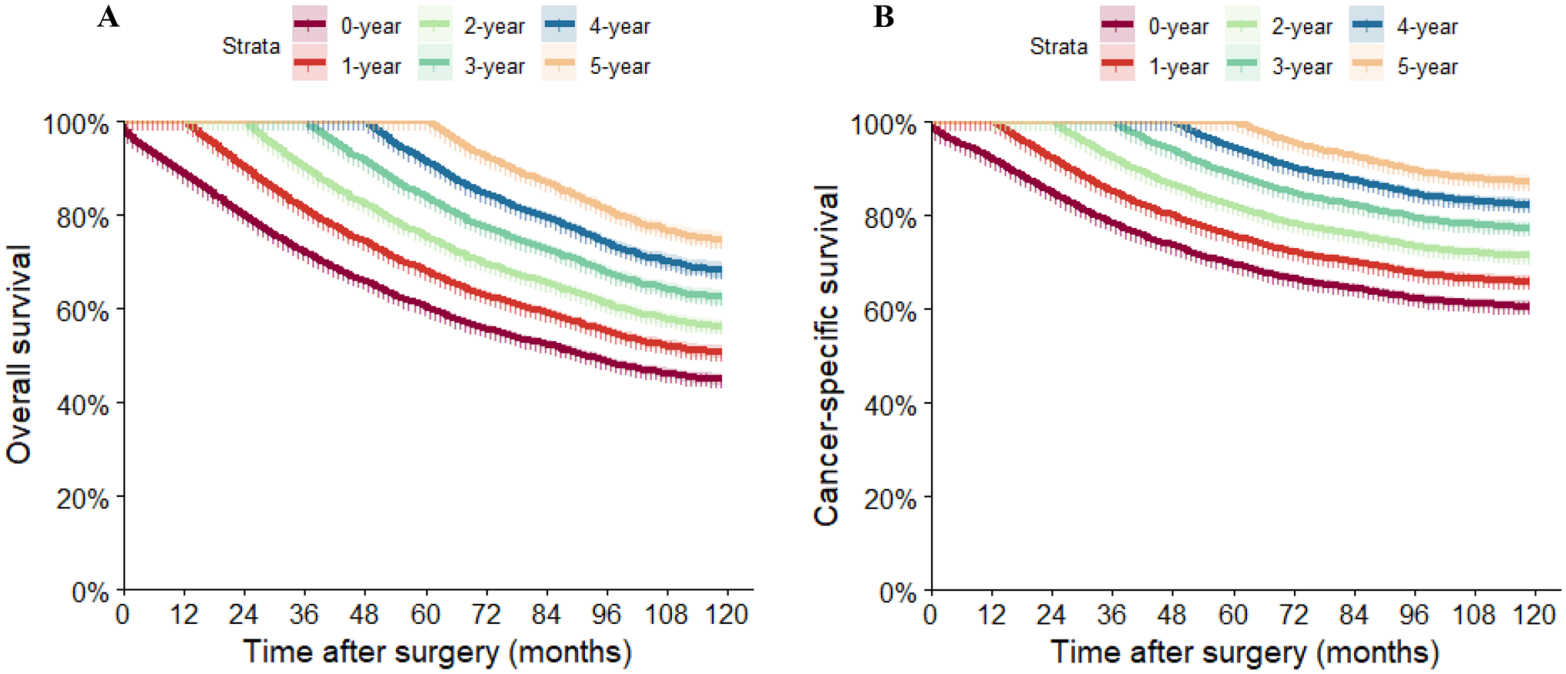
Kaplan–Meier estimates of survival after surgery (0 years) and conditional survival according to years already survived after surgery (1–5 years). **A** Overall survival; **B** cancer-specific survival

The 5-year OS and CSS rates for the patients were 60.2% and 69.5%, respectively. The probabilities of cOS and cCSS are presented in Table 2, and the corresponding survival curves, based on the number of years already survived after surgery, are depicted in Fig. 2. The probability of achieving a 5-year OS after surgery increased progressively from 60.2% immediately after surgery to 67.9%, 75.3%, 83.6%, and 91.4% with 1, 2, 3, and 4 years already survived, respectively. Similarly, the probability of achieving a 5-year CSS after surgery increased from 69.5% directly after surgery to 75.5%, 82.0%, 88.6%, and 94.4% with 1, 2, 3, and 4 years already survived, respectively. These findings highlight that the longer patients had already survived, the greater their chances of additional years of survival [9].

**Table 2:** Conditional overall and cancer-specific survival estimates

		1	2	3	4	5	6	7	8
Overall survival (yrs)	Actuarial survival	Overall survival for patients surviving (yrs)							
1	88.6%								
2	79.9%	90.2%							
3	72.0%	81.3%	90.1%						
4	65.9%	74.4%	82.5%	91.5%					
5	60.2%	67.9%	75.3%	83.6%	91.4%				
6	55.6%	62.8%	69.6%	77.2%	84.4%	92.4%			
7	52.3%	59.0%	65.5%	72.6%	79.4%	86.9%	94.1%		
8	48.8%	55.1%	61.1%	67.8%	74.1%	81.1%	87.8%	93.3%	
9	46.1%	52.0%	57.7%	64.0%	70.0%	76.6%	82.9%	88.1%	94.5%
Cancer-specific survival (yrs)	Actuarial survival	Cancer-specific survival for patients surviving (yrs)							
1	92.0%								
2	84.8%	92.2%							
3	78.4%	85.2%	92.5%						
4	73.6%	80.0%	86.8%	93.9%					
5	69.5%	75.5%	82.0%	88.6%	94.4%				
6	66.4%	72.2%	78.3%	84.7%	90.2%	95.5%			
7	64.4%	70.0%	75.9%	82.1%	87.5%	92.7%	97.0%		
8	62.3%	67.7%	73.5%	79.5%	84.6%	89.6%	93.8%	96.7%	
9	61.2%	66.5%	72.2%	78.1%	83.2%	88.1%	92.2%	95.0%	98.2%

### Risk factors for one-year postoperative cOS and cCSS

Univariate logistic regression analysis revealed associations between age, tumor site, pT stage, pN stage, receipt of chemotherapy, pretreatment CEA levels, number of harvested lymph nodes, presence of tumor deposits, perineural invasion, and marital status with one-year postoperative cOS and cCSS. Sex was found to be associated with one-year postoperative cOS, while radiation was associated with one-year postoperative cCSS. Subsequently, in the multivariate logistic analysis, age, pT stage, pN stage, receipt of chemotherapy, pretreatment CEA levels, number of harvested lymph nodes, presence of tumor deposits, perineural invasion, and marital status were identified as independent risk factors for one-year postoperative cOS and cCSS. Furthermore, sex and tumor site were recognized as independent risk factors for one-year postoperative cOS, while radiation was established as an independent risk factor for one-year postoperative cCSS (Tables 3 and 4).

**Table 3:** Univariable and multivariable Cox proportional hazards analysis of risk factors associated with conditional overall survival

**Variable**	**Univariate analysis**				**Multivariate analysis**			
	HR	95% CI		*P*-value	HR	95% CI		*P*-value
**Age**								
< 50 years	Reference				Reference			
50–59 years	1.299	1.116	1.511	0.001	1.307	1.123	1.522	0.001
60–69 years	1.625	1.411	1.872	< 0.001	1.603	1.390	1.849	< 0.001
70–79 years	2.348	2.044	2.698	< 0.001	2.181	01.892	2.515	< 0.001
80+ years	4.072	3.547	4.676	< 0.001	3.420	2.948	3.967	< 0.001
**Sex**								
Female	Reference				Reference			
Male	1.074	1.002	1.150	0.044	1.248	1.162	1.341	< 0.001
**Race**								
American Indian/Alaska Native	Reference							
Asian or Pacific Islander	0.902	0.601	1.353	0.618				
Black	1.047	0.699	1.567	0.825				
White	0.963	0.650	1.428	0.852				
**Site**								
Ascending colon	Reference				Reference			
Cecum	1.085	0.976	1.207	0.133	0.981	0.881	1.092	0.722
Descending colon	0.776	0.660	0.912	0.002	0.842	0.716	0.992	0.039
Hepatic flexure	1.128	0.953	1.337	0.163	1.119	0.945	1.326	0.193
Rectosigmoid junction	0.811	0.708	0.930	0.003	0.906	0.788	1.041	0.162
Sigmoid colon	0.809	0.728	0.900	< 0.001	0.909	0.816	1.013	0.084
Splenic flexure	0.947	0.775	1.156	0.591	0.947	0.774	1.158	0.594
Transverse colon	0.958	0.833	1.102	0.547	0.931	0.810	1.072	0.320
**pT**								
T3	Reference				Reference			
T4a	1.560	1.428	1.704	< 0.001	1.433	1.311	1.568	< 0.001
T4b	1.756	1.553	1.986	< 0.001	1.524	1.345	1.727	< 0.001
**pN**								
N1a	Reference				Reference			
N1b	1.245	1.136	1.365	< 0.001	1.242	1.133	1.362	< 0.001
N1c	1.287	1.064	1.556	0.009	0.918	0.745	1.130	0.419
N2a	1.543	1.397	1.704	< 0.001	1.610	1.456	1.781	< 0.001
N2b	1.938	1.741	2.156	< 0.001	2.127	1.904	2.376	< 0.001
**Scope of regional lymph nodes**								
1 to 3 regional LNs	Reference							
4 or more regional LNs	1.046	0.739	1.482	0.799				
None	1.169	0.716	1.908	0.533				
**Radiation**								
None/Unknown	Reference							
Yes	1.076	0.923	1.255	0.348				
**Chemotherapy**								
No/Unknown	Reference				Reference			
Yes	0.460	0.429	0.494	< 0.001	0.600	0.555	0.649	< 0.001
**CEA pretreatment**								
CEA negative/normal	Reference				Reference			
CEA positive/elevated	1.544	1.441	1.654	< 0.001	1.392	1.299	1.492	< 0.001
**Harvested lymph nodes**								
< 12LNs	Reference				Reference			
≥ 12LNs	0.630	0.566	0.700	< 0.001	0.609	0.547	0.679	< 0.001
**Tumor deposits**								
No	Reference				Reference			
Yes	1.399	1.283	1.527	< 0.001	1.408	1.277	1.551	< 0.001
**Tumor size**								
< 5 cm	Reference							
≥ 5 cm	1.016	0.948	1.089	0.653				
**Perineural invasion**								
No/unknown	Reference				Reference			
Yes	1.381	1.269	1.502	< 0.001	1.290	1.182	1.408	< 0.001
**Marital status**								
Married	Reference				Reference			
Unmarried	1.349	1.260	1.445	< 0.001	1.195	1.112	1.284	< 0.001

*pT* pathologic tumor, *pN* pathologic nodes, *LNs* lymph nodes, *CEA* carcinoembryonic antigen

**Table 4:** Univariable and multivariable Cox proportional hazards analysis of risk factors associated with conditional cancer-specific survival

**Variable**	**Univariate analysis**				**Multivariate analysis**			
	HR	95% CI		*P*-value	HR	95% CI		*P*-value
**Age**								
< 50 years	Reference				Reference			
50–59 years	1.226	1.044	1.439	0.013	1.231	1.048	1.446	0.011
60–69 years	1.350	1.160	1.572	< 0.001	1.369	1.174	1.596	< 0.001
70–79 years	1.642	1,410	1.912	< 0.001	1.558	1.333	1.820	< 0.001
80+ years	2.529	2.167	2.952	< 0.001	2.200	1.863	2.599	< 0.001
**Sex**								
Female	Reference				Reference			
Male	1.017	0.936	1.105	0.693	1.248	1.162	1.341	< 0.001
**Race**								
American Indian/Alaska Native	Reference							
Asian or Pacific Islander	0.947	0.579	1.550	0.830				
Black	1.135	0.696	1.851	0.610				
White	0.968	0.600	1.562	0.896				
**Site**								
Ascending colon	Reference				Reference			
Cecum	1.248	1.095	1.422	< 0.001	1.110	0.974	1.265	0.119
Descending colon	0.862	0.706	1.053	0.146	0.901	0.737	1.101	0.306
Hepatic flexure	1.199	0.973	1.477	0.087	1.217	0.988	1.499	0.065
Rectosigmoid junction	1.016	0.865	1.194	0.842	1.012	0.847	1.209	0.898
Sigmoid colon	0.897	0.787	1.021	0.100	0.921	0.807	1.051	0.219
Splenic flexure	0.903	0.700	1.165	0.432	0.892	0.691	1.152	0.381
Transverse colon	1.02	0.858	1.214	0.814	0.988	0.830	1.175	0.890
**pT**								
T3	Reference				Reference			
T4a	1.989	1.799	2.199	< 0.001	1.762	1.591	1.951	< 0.001
T4b	2.341	2.042	2.683	< 0.001	1.965	1.711	2.258	< 0.001
**pN**								
N1a	Reference				Reference			
N1b	1.367	1.220	1.532	< 0.001	1.309	1.167	1.468	< 0.001
N1c	1.219	0.956	1.552	0.11	0.829	0.639	1.077	0.160
N2a	1.678	1.484	1.896	< 0.001	1.494	1.312	1.702	< 0.001
N2b	2.388	2.103	2.710	< 0.001	1.881	1.584	2.232	< 0.001
**Scope of regional lymph nodes**								
1 to 3 regional LNs	Reference							
4 or more regional LNs	1.103	0.737	1.649	0.634				
None	1.534	0.893	2.635	0.121				
**Radiation**								
None/unknown	Reference				Reference			
Yes	1.226	1.028	1.461	0.023	1.295	1.059	1.585	0.012
**Chemotherapy**								
No/unknown	Reference				Reference			
Yes	0.581	0.533	0.635	< 0.001	0.652	0.591	0.718	< 0.001
**CEA pretreatment**								
CEA negative/normal	Reference				Reference			
CEA positive/elevated	1.70	1.564	1.847	< 0.001	1.483	1.364	1.613	< 0.001
**Harvested lymph nodes**								
< 12LNs	Reference				Reference			
≥ 12LNs	0.621	0.548	0.705	< 0.001	0.582	0.512	0.662	< 0.001
**Tumor deposits**								
No	Reference				Reference			
Yes	1.509	1.363	1.670	< 0.001	1.481	1.325	1.656	< 0.001
**Tumor size**								
< 5 cm	Reference							
≥ 5 cm	1.066	0.938	0.981	0.132				
**Perineural invasion**								
No/unknown	Reference				Reference			
Yes	1.549	1.403	1.709	< 0.001	1.302	1.175	1.441	< 0.001
**Marital status**								
Married	Reference				Reference			
Unmarried	1.258	1.158	1.367	< 0.001	1.147	1.054	1.247	0.001

*pT* pathologic tumor, *pN* pathologic nodes, *LNs* lymph nodes, *CEA* carcinoembryonic antigen

### Construction and performance of the nomogram

**Fig. 3:**
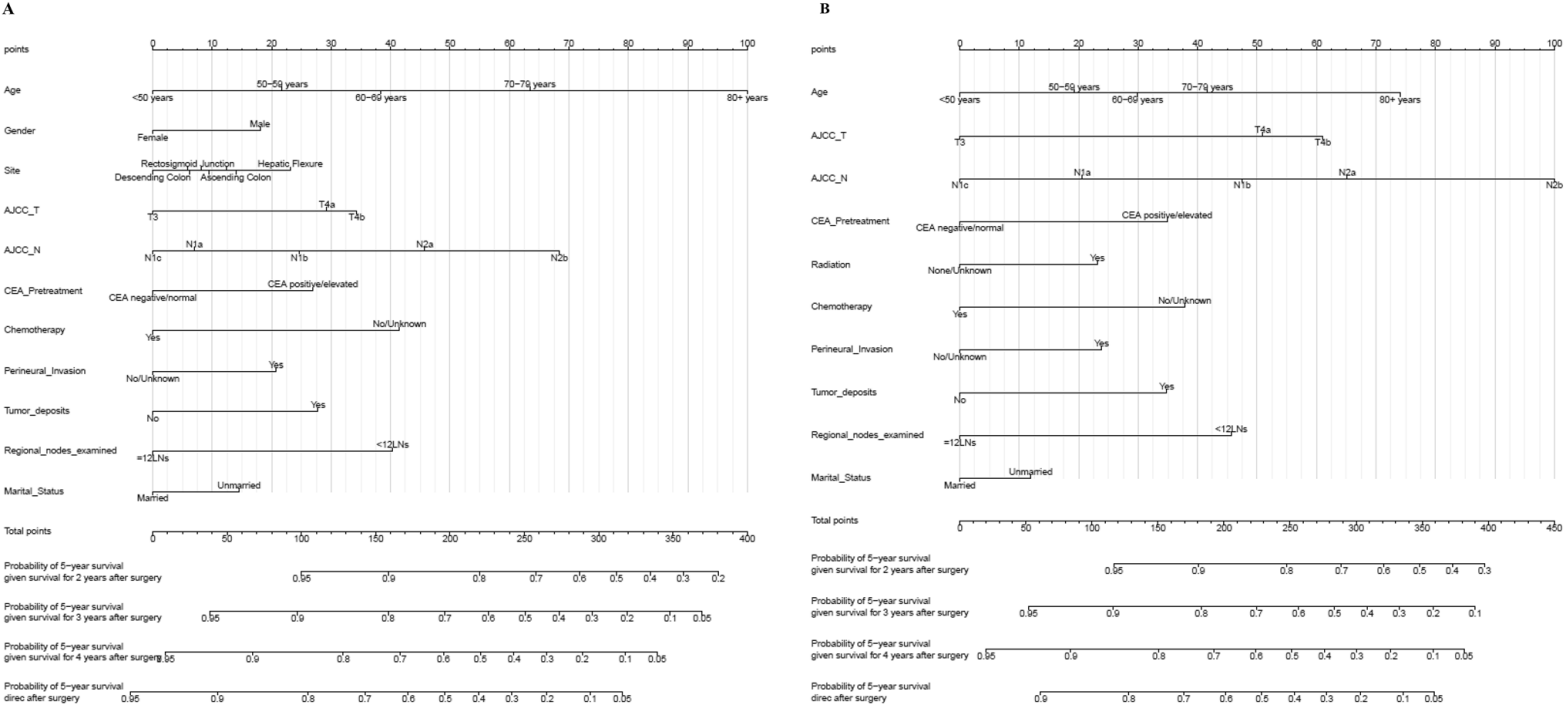
Conditional survival nomogram predicting probability of achieving 5‐year survival after surgery for stage III T3–T4 colon cancer. **A** conditional overall survival (cOS); **B** conditional cancer-specific survival (cCSS)

Predictive nomograms for patients with stage III T3–T4 colon cancer were constructed using independent risk factors identified for one-year postoperative cOS and cCSS, as shown in Fig. 3A, B. These nomograms provide scores corresponding to each risk factor, with the total score representing the sum of all variable scores. The risk of developing cOS and cCSS at one year postoperatively is determined by drawing a line from the total score to the corresponding risk score. In the training cohort, the nomogram had a one-year postoperative cOS *C*-index of 0.701 (95% CI, 0.711–0.691) and a one-year postoperative cCSS *C*-index of 0.701 (95% CI, 0.713–0.689). After validation, the C-index was 0.707 (95% CI, 0.721–0.693) for one-year postoperative cOS and 0.700 (95% CI, 0.716–0.684) for one-year postoperative cCSS. These results indicate that the nomogram model has strong predictive performance and reliability.

**Fig. 4:**
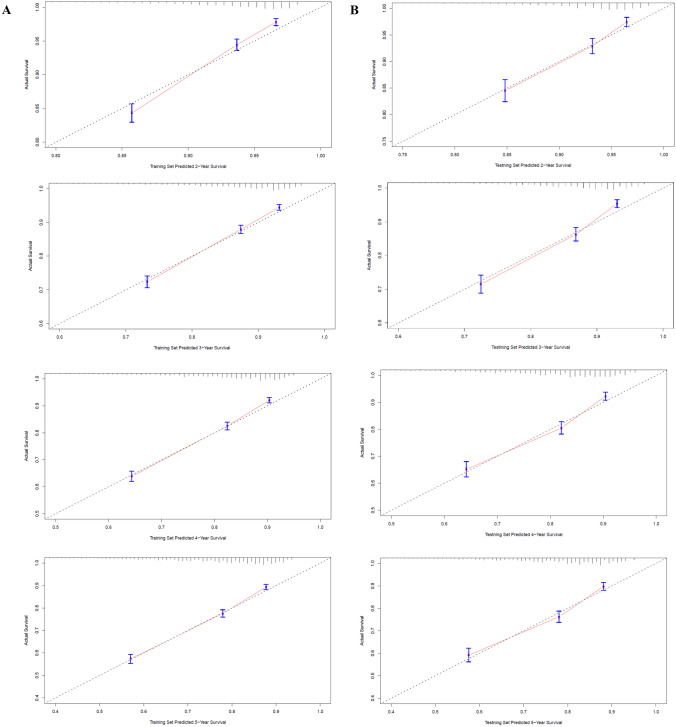
Calibration curves of nomograms for conditional overall. **A** Calibration curve of 2-year, 3-year, 4-year, and 5-year cOS in the training cohort. **B** Calibration curve of 2-year, 3-year, 4-year, and 5-year cOS in the validation cohort

**Fig. 5:**
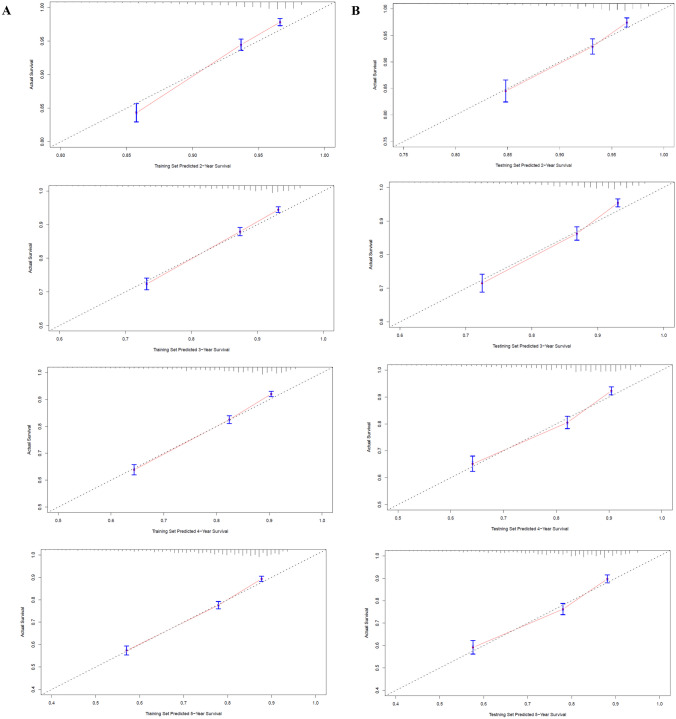
Calibration curves of nomograms for conditional cancer-specific survival. **A** Calibration curve of 2-year, 3-year, 4-year, and 5-year cCSS in the training cohort. **B** Calibration curve of 2-year, 3-year, 4-year, and 5-year cCSS in the validation cohort

**Fig. 6:**
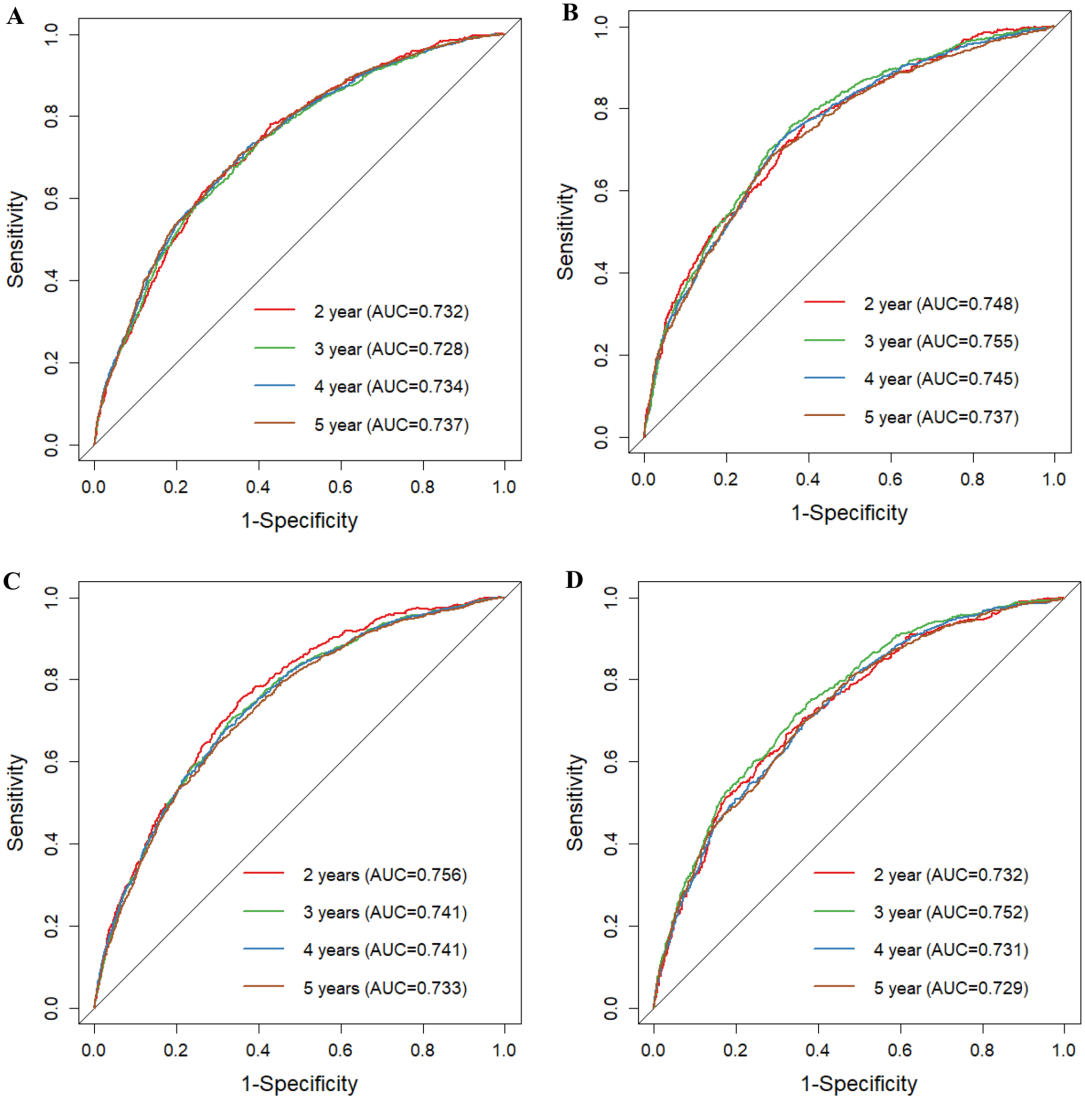
ROC curves of nomogram for predicting conditional overall and cancer-specific survival. **A** ROC curve of cOS in the training cohort. **B** ROC curve of cOS in the validation cohort. **C** ROC curve of cCSS in the training cohort. **D** ROC curve of cCSS in the validation cohort

**Fig. 7:**
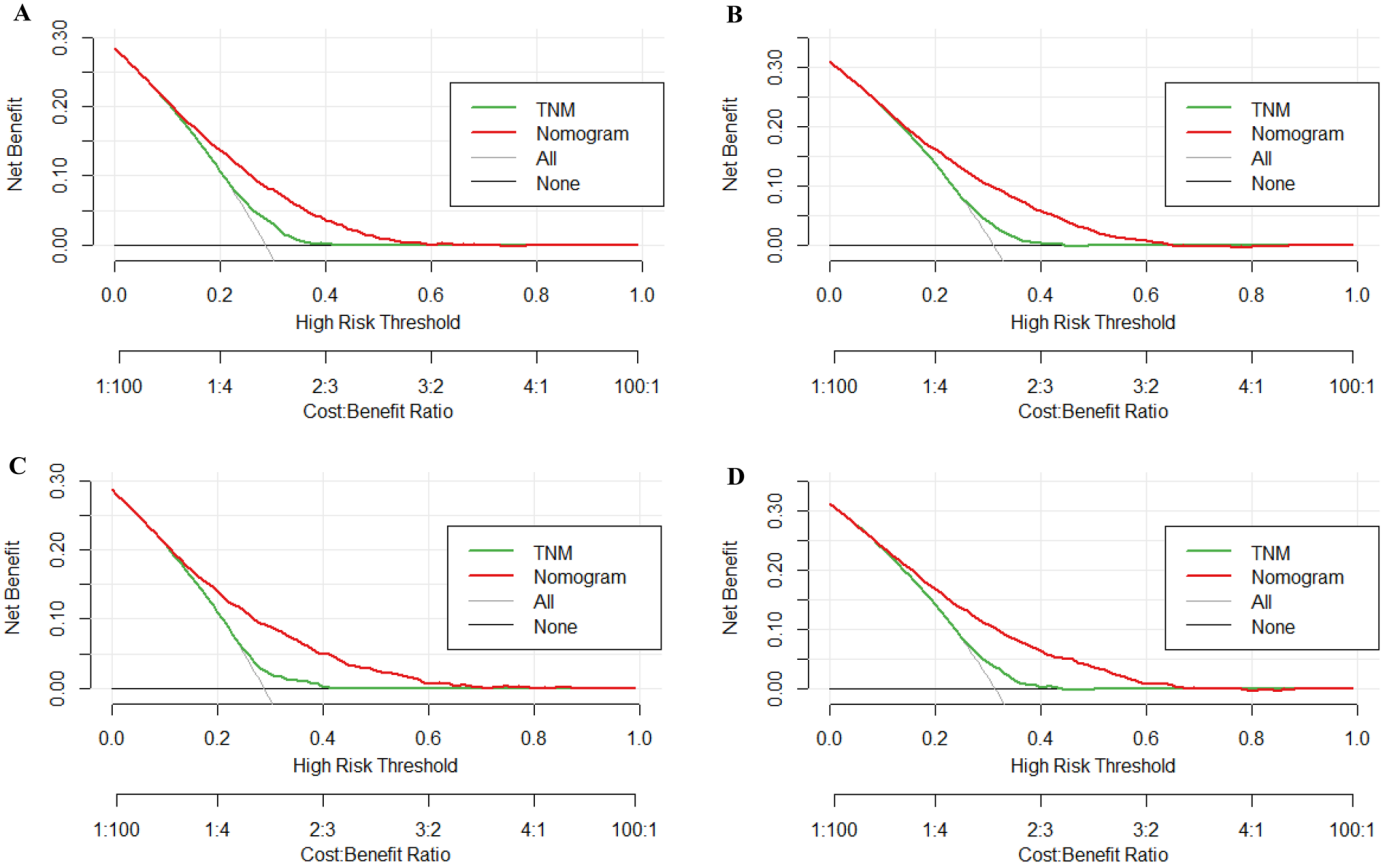
The decision curve analysis (DCA) curves of nomograms for conditional overall and cancer-specific survival, the nomograms (red line) had a better clinical net value than the TNM staging system (green line). **A** DCA curve of cCSS in the training cohort. **B** DCA curve of cCSS in the validation cohort. **C** DCA curve of cOS in the training cohort. **D** DCA curve of cOS in the validation cohort

As indicated by the calibration curves, the nomograms demonstrated a substantial alignment between predicted and observed outcomes in both the training and validation cohorts, with prediction curves closely resembling the diagonal line (Figs. 4 and 5). In the training and validation cohorts, the 2-year, 3-year, 4-year, and 5-year AUC values for one-year postoperative conditional overall survival (cOS) were 0.732, 0.728, 0.734, and 0.737 and 0.748, 0.755, 0.745, and 0.737, respectively (Fig. 6A, B). Similarly, in the training and validation cohorts, the 2-year, 3-year, 4-year, and 5-year AUC values for one-year postoperative cCSS were 0.732, 0.728, 0.734, and 0.737 and 0.748, 0.755, 0.745, and 0.737, respectively (Fig. 6C, D). Decision curve analysis revealed that, when compared to the AJCC TNM staging system, the nomograms achieved a superior net benefit in predicting all-cause and cancer-specific mortality in both the training and validation cohorts (Fig. 7).

## Discussion

Stage III colon cancer is characterized by lymph node metastasis, with T3–T4 tumors often deeply infiltrating the colon wall and nearby lymph nodes, significantly reducing the chances of survival [1]. Therefore, the risk of postoperative death or recurrence in colon cancer patients is not constant [14]. For patients who have survived for some time after surgery, the assessment of prognosis based on OS or CSS immediately after surgery may lead to an underestimation of survival, necessitating frequent follow-up monitoring. In this study, we evaluated the 1-year cOS and cCSS after radical resection in patients with stage III T3–T4 colon cancer. Additionally, we developed a nomogram designed to provide precise prognosis information to both patients and physicians. This nomogram allows for a visual representation of the increasing likelihood of surviving stage III T3–T4 colon cancer as more time passes. The longer a patient survives after surgery, the greater their chance of surviving for one year. Consequently, this study aims to assess the CS of stage III T3–T4 colon cancer patients who undergo radical surgery. Our findings demonstrate that the chances of survival increase significantly with a longer post-surgery survival period. We have created CS nomogram plots to offer acolon cancerurate prognostic information for both patients and clinicians.

Conditional survival incorporates the time a patient has already survived into survival probability assessments. In this study, if patients have survived for 3 years postoperatively, the probabilities of achieving a 5-year OS and CSS have increased from 60.2% and 69.5% immediately post-surgery to 83.6% and 88.6%, respectively. Therefore, CS can offer valuable supplementary information for predicting post-radical surgery survival in patients with stage III T3–T4 colon cancer. This trend is supported by CS studies in other malignancies [15, 16]. Furthermore, with increasing survival time, patients with poorer tumor characteristics experience a more significant improvement in CS compared to those with better characteristics. Most high-risk patients with malignancies sucolon cancerumb shortly after surgery.

In this analysis, age, pT stage, pN stage, chemotherapy, pretreatment CEA levels, number of harvested lymph nodes, tumor deposits, perineural invasion, and marital status were identified as independent risk factors for one-year postoperative cOS and cCSS. Additionally, sex and site were identified as independent risk factors for one-year postoperative cOS, while radiation was identified as an independent risk factor for one-year postoperative cCSS. pT stage, pN stage, chemotherapy, pretreatment CEA levels, perineural invasion have been widely recognized in numerous studies as independent risk factors affecting tumor survival [17, 18]. Shimomura’s study concluded that adequate lymph node examination is essential to ensure the prognostic value of the lymph node ratio in patients with stage III colorectal cancer [19]. Lewis et al. found that older patients diagnosed with stage I or II disease had worse overall survival, but at higher stages of the disease, overall survival for all age groups was similar [20]. Krajc et al. demonstrated that marriage was associated with significantly higher overall survival, while unmarried men were associated with significantly lower survival rates, marital status should be considered when providing cancer care [21]. Pu et al. concluded that stage N1 patients with tumor deposits have the same risk of recurrence as stage N2 patients without tumor deposits, highlighting tumor deposition as an independent poor prognostic factor, particularly in stage N2 [22]. Liu et al. [23] developed nomograms for predicting the overall survival of stage II-III colorectal cancer patients. They also identified preoperative mean platelet volume, preoperative platelet distribution width, monocytes, and postoperative adjuvant chemotherapy as independent risk factors for survival in stage II–III rectal cancer. These findings collectively underscore the significance of these risk factors in predicting postoperative survival outcomes in patients with colon and rectal cancer.

While radiotherapy is an important clinical option for colorectal cancer, its utilization is constrained by the low radiosensitivity of colorectal cancer and the high toxicity to surrounding normal tissues [24]. The choice of dosage and irradiation range is of paramount importance regarding its impact on normal tissues. Precise control is imperative in the planning of radiation therapy to minimize radiation exposure to normal tissues. Modern radiation therapy techniques are typically designed to reduce damage to normal tissues while maximizing the impact on tumor tissues [25]. In this study, postoperative radiotherapy is a potential risk factor for stage III T3–T4 colon cancer patients. This may be attributed to the inherent nature of radiotherapy, which can potentially cause harm to normal tissues. McLaughlin et al. contend that adjuvant radiotherapy is not routinely employed for deterministic treatment of T4 non-rectal colon adenocarcinoma and may also lead to long-term effects, including permanent tissue damage, such as intestinal or urinary tract strictures, as well as an increased risk of subsequent malignancies [26].

As survival time increases, CS nomograms can offer more acolon cancerurate prognostic predictions for survivors of stage III T3–T4 colon cancer following surgical resection, compared to traditional static survival assessment methods. At each follow-up interval, survivors can acolon canceress real-time modified survival estimates based on their acolon cancerrued survival time. Understanding the increasing likelihood of survival over time can help alleviate anxiety among survivors and improve their quality of life, especially for those initially diagnosed with a poor prognosis. Clinical practitioners can utilize CS nomograms to assess the risk of death or recurrence, enabling them to design effective follow-up and monitoring strategies. This approach, rooted in the “Conditional Survival Analysis of Stage III T3–T4 Colon Cancer One Year Following Surgical Resection,” provides valuable insights into the dynamic nature of postoperative survival and empowers both patients and clinicians with better-informed decisions.

This study does have some limitations: (1) Due to the lack of external validation, our analyses may be subject to survival bias, which may lead to an overestimation of survival because we focused only on the survival time of survivors. To reduce this bias in future studies, we recommend a rigorous approach that includes external validation, ROC curve analysis over time, and inverse probability weighting. These strategies are essential to more accurately assess and report conditional survival, ensure the reliability of study results, and deepen understanding of the impact of treatment on survival outcomes. (2) This study applies primarily to non-specific adenocarcinoma types and may not be applicable to all adenocarcinoma subtypes, especially mucinous and imprinted cell carcinomas. Future studies should consider the inclusion of these subtypes to provide broader applicable insights. (3) The current SEER database lacks detailed information on treatment modalities, including perioperative management and specific chemotherapy regimens, and this lack of information prevents us from fully assessing the impact of various treatments on survival.

## Conclusion

We developed nomograms and predictive models designed to predict survival in patients with postoperative stage III T3–T4 colon cancer. Although our models are expected to provide more accurate estimates of conditional survival, our analyses were subject to survival bias. This may lead to an overestimation of conditional survival and may affect the generalizability of our findings. Future studies should seek to externally validate and refine these predictive models to ensure that they provide appropriate and accurate tools for patient risk assessment.

## Data Availability

The data used and/or analyzed during the current study are available from the corresponding authors on reasonable request.

## References

[1] Li C et al (2018) Survival nomograms for stage III colorectal cancer. Medicine (Baltimore) 97(49):e13239. 10.1097/MD.0000000000013239 30544384 10.1097/MD.0000000000013239 PMC6310595

[2] Zheng X, Cen W, Zhu J, Ye L (2023) Prognostic value of tumor deposits in stage III colorectal cancer patients with different N stages: a population-based, retrospective, cohort study. Ann Surg Oncol 30(13):8067–8073. 10.1245/s10434-023-14338-x 37782414 10.1245/s10434-023-14338-x

[3] Li C, Pei Q, Zhu H, Tan F (2018) Survival nomograms for stage III colorectal cancer. Medicine (Baltimore) 97(49):e13239. 10.1097/MD.0000000000013239 30544384 10.1097/MD.0000000000013239 PMC6310595

[4] Dekker YW, Peeters KC, Putter H et al (2010) Metastatic lymph node ratio in stage III rectal cancer; prognostic significance in addition to the 7th edition of the TNM classification. Eur J Surg Oncol 36:1180–6 20884164 10.1016/j.ejso.2010.09.007

[5] Fokas E, Fietkau R, Hartmann A et al (2018) Neoadjuvant rectal score as individual-level surrogate for disease-free survival in rectal cancer in the Cao/Aro/Aio-04 randomized phase iii trial. Ann Oncol 7:1521e7 10.1093/annonc/mdy143 29718095

[6] Karagkounis G, Liska D, Kalady MF (2019) Conditional probability of survival after neoadjuvant chemoradiation and proctectomy for rectal cancer: what matters and when. Dis Colon Rectum 1:33e9 10.1097/DCR.0000000000001239 30451761

[7] Ren D et al (2022) Development and internal validation of a nomogram-based model to predict three-year and five-year overall survival in patients with stage II/III COLON CANCER. Cancer Manag Res 14(14):225–236. 10.2147/CMAR.S335665 35058717 10.2147/CMAR.S335665 PMC8765714

[8] Camp RL, Dolled-Filhart M, Rimm DL (2004) X-tile: a new bio-informatics tool for biomarker assessment and outcome-based cut-point optimization. Clin Cancer Res 10(21):7252–7259 15534099 10.1158/1078-0432.CCR-04-0713

[9] Zheng Z, Wang X, Liu Z, Lu X, Huang Y, Chi P (2021) Individualized conditional survival nomograms for patients with locally advanced rectal cancer treated with neoadjuvant chemoradiotherapy and radical surgery. Eur J Surg Oncol 47(12):3175–3181 34120806 10.1016/j.ejso.2021.06.010

[10] Dikken JL, Baser RE, Gonen M et al (2013) Conditional probability of survival nomogram for 1-, 2-, and 3-year survivors after an R0 resection for gastric cancer. Ann Surg Oncol 5:1623e30 10.1245/s10434-012-2723-6 PMC4091759 23143591

[11] Feliu J, Espinosa E, Basterretxea L, Paredero I, Llabrés E, Jiménez-Munárriz B et al (2021) Prediction of chemotoxicity, unplanned hospitalizations and early death in older patients with colorectal cancer treated with chemotherapy. Cancers 14(1):127. 10.3390/cancers14010127 35008291 10.3390/cancers14010127 PMC8749992

[12] Stephenson AJ, Scardino PT, Eastham JA et al (2005) Postoperative nomogram predicting the 10-year probability of prostate cancer recurrence after radical prostatectomy. J Clin Oncol 28:7005e12 10.1200/JCO.2005.01.867 PMC2231088 16192588

[13] Liu Y, Wang J, Li L, Qin H, Wei Y, Zhang X et al (2022) AC010973.2 promotes cell proliferation and isone of six stemness-related genes that predict overall survival of renal clearcell carcinoma. Scientific Rep 12(1):4272. 10.1038/s41598-022-07070-1 10.1038/s41598-022-07070-1 PMC8917182 35277527

[14] Hyuna Sung, Jacques Ferlay, Rebecolon cancera L. Siegel. Global Cancer Statistics (2020) GLOBOCAN estimates of incidence and mortality worldwide for 36 cancers in 185 countries. CA Cancer J Clin 2021(71):209–249 10.3322/caac.21660 33538338

[15] Mayo SC, Nathan H, Cameron JL et al (2012) Conditional survival in patients with pancreatic ductal adenocarcinoma resected with curative intent. Cancer. 10:2674e81 10.1002/cncr.26553 PMC3578343 21935914

[16] Hagens E, Feenstra ML, Eshuis WJ et al (2020) Conditional survival after neoadjuvant chemoradiotherapy and surgery for oesophageal cancer. Br J Surg 8:1053e61 10.1002/bjs.11476 PMC7317937 32017047

[17] Liao Z (2023) A competing risk nomogram to predict cancer-specific mortality of patients with late-onset colorectal cancer. J Cancer Res Clin Oncol. 10.1007/s00432-023-05069-3 37548769 10.1007/s00432-023-05069-3 PMC11798212

[18] O’Sullivan DE, Sutherland RL (2022) Risk factors for early-onset colorectal cancer: a systematic review and meta-analysis. Updat Surg 20(6):1229-1240.e5. 10.1016/j.cgh.2021.09.052 10.1016/j.cgh.2021.01.037 33524598

[19] Shimomura M et al (2011) Adequate lymph node examination is essential to ensure the prognostic value of the lymph node ratio in patients with stage III colorectal cancer. Surg Today 41(10):1370–1379. 10.1007/s00595-010-4446-2 21922359 10.1007/s00595-010-4446-2

[20] Lewis SL (2023) Association of age and overall survival in surgically resected colorectal cancer patients. J Surg Res 281:321–327. 10.1016/j.jss.2022.08.031. Epub 2022 Oct 11. 36240718 10.1016/j.jss.2022.08.031

[21] Krajc K et al (2023) Marital status and survival in cancer patients: a systematic review and meta-analysis. Cancer Med 12(2):1685–1708. 10.1002/cam4.5003 35789072 10.1002/cam4.5003 PMC9883406

[22] Pu H et al (2022) Significance of tumor deposits combined with lymph node metastasis in stage III colorectal cancer patients: a retrospective multi-center cohort study from China. Int J Colorectal Dis 37(6):1411–1420 35595975 10.1007/s00384-022-04149-z PMC9167180

[23] Liu J, Huang X et al (2020) A nomogram for predicting overall survival in stage II-III colorectal cancer. Updat Surg 9(7):2363–2371. 10.1002/cam4.2896. Epub 2020 Feb 6. 10.1002/cam4.2896 PMC7131840 32027098

[24] Tan G, Lin C et al (2022) Gasdermin E regulation of radiosensitivity in colorectal cancer and radiation-induced gut damages. Cancer Lett 31(529):1–10. 10.1016/j.canlet.2021.12.034. Epub 2021 Dec 31. 10.1016/j.canlet.2021.12.034 34979164

[25] Whelan S, Burneikis D, Kalady MF (2022) Maximizing local control and minimizing toxicity in rectal cancer. J Surg Oncol 125(1):46–54. 10.1002/jso.26743 34897711 10.1002/jso.26743

[26] McLaughlin C, Kim NK, Bandyopadhyay D et al (2019) Adjuvant radiation therapy provides a cause-specific survival advantage for T4 non-rectal colon adenocarcinoma: an analysis of the SEER database. Radiother Oncol 133:50–53. 10.1016/j.radonc.2018.11.026 30935581 10.1016/j.radonc.2018.11.026 PMC10105524

